# Circulating CXCR5^+^CD4^+^ T Follicular-Like Helper Cell and Memory B Cell Responses to Human Papillomavirus Vaccines

**DOI:** 10.1371/journal.pone.0137195

**Published:** 2015-09-02

**Authors:** Ken Matsui, Joseph W. Adelsberger, Troy J. Kemp, Michael W. Baseler, Julie E. Ledgerwood, Ligia A. Pinto

**Affiliations:** 1 Human Papillomavirus (HPV) Immunology Laboratory, Leidos Biomedical Research, Inc., Frederick National Laboratory for Cancer Research, Frederick, Maryland, United States of America; 2 AIDS Monitoring Laboratory, Clinical Service Program, Applied and Developmental Directorate, Leidos Biomedical Research, Inc., Frederick National Laboratory for Cancer Research, Frederick, Maryland, United States of America; 3 Vaccine Research Center, National Institute of Allergy and Infectious Diseases, National Institutes of Health, Bethesda, Maryland, United States of America; Instituto Butantan, BRAZIL

## Abstract

Through the interaction of T follicular helper (Tfh) cells and B cells, efficacious vaccines can generate high-affinity, pathogen-neutralizing antibodies, and memory B cells. Using CXCR5, CXCR3, CCR6, CCR7, PD1, and ICOS as markers, Tfh-like cells can be identified in the circulation and be classified into three functionally distinct subsets that are PD1^+^ICOS^+^, PD1^+^ ICOS^-^, or PD1^-^ICOS^-^. We used these markers to identify different subsets of CXCR5^+^CD4^+^ Tfh-like cells in response to highly immunogenic and efficacious vaccines for human papillomaviruses (HPV): Cervarix and Gardasil. In this small study, we used PBMC samples from 11 Gardasil recipients, and 8 Cervarix recipients from the Vaccine Research Center 902 Study to examine the induction of circulating Tfh-like cells and IgD^-^CD38^Hi^CD27^+^ memory B cells by flow cytometry. PD1^+^ICOS^+^ CXCR3^+^CCR6^-^CXCR5^+^CD4^+^ (Tfh1-like) cells were induced and peaked on Day (D) 7 post-first vaccination, but not as much on D7 post-third vaccination. We also observed a trend toward increase in PD1^+^ICOS^+^ CXCR3^-^CCR6^-^CXCR5^+^CD4^+^ (Tfh2-like) cells for both vaccines, and PD1^+^ICOS^+^ CXCR3^-^CCR6^+^CXCR5^+^CD4^+^ (Tfh17-like) subset was induced by Cervarix post-first vaccination. There were also minimal changes in the other cellular subsets. In addition, Cervarix recipients had more memory B cells post-first vaccination than did Gardasil recipients at D14 and D30. We found frequencies of memory B cells at D30 correlated with anti-HPV16 and 18 antibody titers from D30, and the induction levels of memory B cells at D30 and PD1^+^ICOS^+^Tfh1-like cells at D7 post-first vaccination correlated for Cervarix. Our study showed that induction of circulating CXCR5^+^CD4^+^ Tfh-like subsets can be detected following immunization with HPV vaccines, and potentially be useful as a marker of immunogenicity of vaccines. However, further investigations should be extended to different cohorts with larger sample size to better understand the functions of these T cells, as well as their relationship with B cells and antibodies.

## Introduction

Highly efficacious vaccines can generate high-affinity, pathogen neutralizing antibodies that could persist for years in all recipients. It is also essential that immunization with such vaccines leads to the generation of class-switched, antibody-secreting long-lived plasma cells, as well as the generation of memory B cells to provide protection from pathogens [1,2]. Such humoral immune responses require the interaction of B lymphocytes and a specialized subset of CD4^+^ T-helper (Th) cells, T follicular helper (Tfh) cells, in secondary lymphoid tissues [1,3–7]. The Tfh/B cell interaction, through which provision of help is delivered to a B cell from a Tfh cell, is critical for the development of germinal centers (GC), in which class-switching, affinity maturation, and generation of long-lived plasma cells and memory B cells occur [1,3–7]. Therefore, monitoring the induction of Tfh cells, memory B cells, and development of antigen-specific antibodies, may provide further insights into the immunogenicity and efficacy of vaccines.

Although *bona fide* Tfh cells are found in secondary lymphoid organs [8–10], studies have shown that Tfh-like cells can be detected within the C-X-C chemokine receptor (CXCR) 5-expressing CD4^+^ T cells in the blood [3,4,11]. In addition to CXCR5, program cell death (PD) 1, inducible T-cell costimulator (ICOS), and C-C chemokine receptor (CCR) 7 can be used to identify these circulating CXCR5^+^CD4^+^ Tfh-like cells within the CD45RO^+^ CD4^+^ T cell population [3,12,13]. Although these circulating CXCR5^+^CD4^+^ cells differ from the lymphoid Tfh cells in their gene expression profiles, and that they do not express much Bcl-6 protein, functionally, these circulating CXCR5^+^CD4^+^ cells have been shown to provide help to B cells [11,13–18]. Furthermore, circulating CXCR5^+^CD4^+^ cells can be divided into different cellular subsets by using CCR6 and CXCR3 as markers [14]. Based on the expression of these markers, circulating CXCR5^+^CD4^+^ cells can be categorized as Tfh1-like (CXCR3^+^CCR6^-^), Tfh2-like (CXCR3^-^CCR6^-^), and Tfh17-like (CXCR3^-^CCR6^+^) cells. It was also reported that CXCR3^-^ Tfh2-like and Tfh17-like cells can provide help to naive B cells to become antibody-secreting cells *in vitro*, while CXCR3^+^ Tfh1-like cells could not provide help [14]. However, in response to influenza vaccine, CXCR3^+^ Tfh1-like cells transiently expressed ICOS, along with PD1, which peaked on day 7 post-vaccination, and that these cells were able to provide help to memory B cells [16,18]. Bentebibel *et al* also reported that the frequency of these cells positively correlated with the levels of influenza-specific antibodies and induction levels of plasmablasts [18]. He *et al* reported that CXCR3^+^ Tfh1-like cells were induced in response to ovalbumin using Sigma Adjuvant System but not with alum in mice [16], indicating that an adjuvant could potentially influence the induction of particular Tfh-like subsets. Locci *et al* and Boswell *et al* showed that CXCR3^-^ PD1^+^ ICOS^-^ Tfh-like cells had the transcriptional profiles that closely resembled those of Tfh cells from GC in tonsils [15,17]. Locci *et al* showed that the presence of these cells correlated with the titers of broadly neutralizing HIV antibodies, and were most efficient at activating memory B cells to become IgG-secreting cells *in vitro* [15]. However, Boswell *et al* have reported that there was no correlation between the Tfh-like cell subset and the IgG^+^ B cells [17]. Furthermore, Chevalier *et al* also showed that circulating CXCR5^+^ CD4^+^ cells have the capacity to provide help to B cells, but the phenotype of these T cells did not resemble that of *bona fide* Tfh cells [11]. Although further work is still needed to understand about the circulating CXCR5^+^ CD4^+^ Tfh-like cells, and the relationship between these Th cells, antibodies, and memory B cells, these studies can be used as a framework to study immune responses in different systems. Currently, based on the expression of CXCR5, CXCR3, CCR6, CCR7, PD1, and ICOS, nine different subsets of circulating CXCR5^+^CD4^+^ Tfh-like cells can be identified [19]. In the current study, we evaluated changes in these subsets in response to vaccination with the HPV vaccines.

Human papillomaviruses (HPV) 16 and18 are the most common and clinically relevant high-risk types or strains, and these two types are responsible for approximately 70% of cervical cancer cases [20,21]. The two currently licensed prophylactic HPV vaccines, Gardasil and Cervarix, contain the major structural capsid protein late 1 (L1)-based virus-like-particles (VLP) of HPV16 and 18. In addition, Gardasil also contains L1 VLPs of HPV6 and 11, the two major types responsible for genital warts [21–23]. While Gardasil is adjuvanted by aluminum hydroxyphosphate sulfate, Cervarix employs Adjuvant System 04 (AS04), which contains a Toll-like receptor (TLR) 4 agonist 3-O-desacyl-4-monophosphoryl lipid A (MPL) plus aluminum salt. For both vaccines, 3-immunization schedule has traditionally been recommended [21,22].

Both vaccines have demonstrated excellent efficacy against HPV types that are included in the vaccines with induction of anti-HPV16 and-HPV18 antibodies, which have persisted in serum for years after vaccination [21,22,24]. While there has been no formal identification of correlates of protection, and that the protective levels of antibody titers have not been defined, it is believed that HPV-specific antibodies play a critical role in providing protection from infections [25]. Although both vaccines are efficacious, there have been studies that reported differences in the immune responses to these two vaccines. Anti-HPV16 and-HPV18 antibody levels have been shown to be higher in those who received Cervarix [26–28]. Also, the number of HPV16- and HPV18-specific memory B cells has been shown to be higher in the Cervarix recipients, as measured by ELISPOT assay [26,27]. In addition, the adjuvant system employed is different for the two vaccines, which may influence the types of Tfh cell responses, leading to differences in antibody titers and memory B cell formation.

Because of the success of the HPV vaccines, they represent an ideal model to dissect cellular and molecular mechanisms of immunogenicity and efficacy against HPV. In this study, we examined if there are differences in the levels of circulating CXCR5^+^CD4^+^ Tfh-like subsets and memory B cell responses between the two HPV vaccines, and whether responses by these circulating CXCR5^+^CD4^+^ cells may be used as a marker of immunogenicity. Furthermore, we examined how these immune responses might correlate to the induction of antigen-specific antibodies. In this longitudinal analysis, we used peripheral blood mononuclear cells (PBMC) of female volunteers aged 18–25 who had received either Gardasil or Cervarix in the Vaccine Research Center (VRC) 902 Study [28] to monitor the responses of different subsets of circulating CXCR5^+^CD4^+^ cells and IgD^-^ CD38^Hi^ CD27^+^memory B cells at several time points post-vaccination. This VRC study was chosen because it is one of the few HPV vaccine trials, if any, that collected peripheral blood samples at multiple time points early post-vaccination that included day 7 post-immunization samples. We examined the frequencies of different CXCR5^+^CD4^+^ Tfh-like cellular subsets and memory B cells over time, and investigated the relationships among these lymphocytes and HPV16 and 18-specific IgG.

## Materials and Methods

### Study populations and sample collection

PBMC and serum samples from the VRC 902 Study (ClinicalTrial.gov: #NCT01132859), National Institute of Allergy and Infectious Diseases (NIAID) were used in the current study. The details of the VRC 902 Study have been described elsewhere [28]. Briefly, 18–25 year old (mean and median age 23) female volunteers were enrolled into this clinical trial. This study was conducted by the Vaccine Research Center (VRC), NIAID, at the National Institutes of Health Clinical Center, Bethesda, Maryland, USA. The study, including the consent procedure, was reviewed and approved by the NIAID Institutional Review Board. The study team followed human experimental guidelines for conducting clinical research from the US Department of Health and Human Services and in accordance with principles expressed in the Declaration of Helsinki. All subjects gave written informed consent prior to participation; the original signed consent form was filed in the subject’s medical record and a copy of the signed consent form was given to the participant. This was a two-arm study, in which twenty-seven volunteers were randomized to receive either the scheduled three-dose regimen of Gardasil (Merck & Co.; *n* = 15) or Cervarix (GlaxoSmithKline; *n* = 12) in the deltoid muscle [21,22]. Gardasil contains 20, 40, 40, and 20 μg of VLP for HPV6, 11, 16, and 18, respectively [21,22]. Cervarix contains 20 μg each of VLP for HPV16 and 18 [21,22]. Based on the self-reporting survey, these women had not received any HPV vaccines prior to the study. At various time points, both prior to and post vaccinations, peripheral blood samples were collected, and sera and PBMCs were isolated [29].

PBMC samples from influenza vaccine recipients were used to optimize and validate the staining of cells to identify different subsets of circulating CXCR5^+^CD4^+^ cells. Five influenza vaccine (Fluvirin, Novartis) recipients (one male and four females, median age 44, range 26–50) were recruited through the Occupational Health Services Research Donor Program at Frederick National Laboratory for Cancer Research, Leidos Biomedical Research Inc. (Frederick, MD). Whole blood samples were collected from these individuals prior to immunization (Day 0), and on 6–7 days post-vaccination, and PBMC were isolated through Histopaque-1077 (Sigma Aldrich, St. Louis, MO) [30].

### Immunization schedule and sample selection

Vaccination schedules were month (M) 0, M1, and M6 for Cervarix, and M0, M2, and M6 for Gardasil (S1A Fig). The overall summary of the days at which the samples were collected for the tested subjects is shown in S1 Table. We tested PBMC samples from the following time points: pre-vaccination, days 1, 7, 14, and 30 post-first vaccination (S1B Fig). Also, samples from M6 (pre-third vaccination), days 2 and 7 post-third vaccination, and M7 were tested (S1B Fig). PBMC samples from Day 0 (just prior to the first vaccination) were not available for the current study. Therefore, the samples from the pre-vaccination time points were used to represent specimens for the baseline time point. Because the overall availability of the PBMC samples was limited, we could not test all 27 subjects. As a result, we tested 11 and 8 subjects from Gardasil and Cervarix, respectively. Furthermore, participants who showed up on the follow-up visits were limited for the third vaccination time period.

### Antibodies and reagents for flow cytometry

Flow cytometry was performed on PBMCs from both the influenza vaccine immunized volunteers and those from the VRC 902 Study. There were 2 panels of antibodies; one for CXCR5^+^CD4^+^ T cell subsets, and the second panel for memory B cells. For the staining of T cells, the following antibodies were used: CXCR5-AF488 (RF8B2, BD Biosciences, San Diego CA), PD1-PE (eBioJ105, eBioscience, San Diego, CA), CD45RO-PE/CF594 (UCHL1, BD Biosciences), CCR6-PerCP/Cy5.5 (G034E3, Biolegend, San Diego, CA), ICOS-PE/Cy7 (C398.4A, Biolegend), CCR7-AF647 (3D12, BD Biosciences), CXCR3-AF700 (1C6/CXCR3, BD Biosciences), CD4-APC/eFluor 780 (SK3, eBioscience), CD27-V450 (M-T271, BD Biosciences), CD8-BV510 (SK1, BD Biosciences), and CD3-BV605 (SK7, BD Biosciences). For B cells, IgD-AF488 (IA6-2, Biolegend), CD19-PE (HIB19, Biolegend), CD38-PE/Cy7 (HB-7, Biolegend), and CD27-BV450 (M-T271, BD Biosciences) were used. To exclude dead cells, Zombie UV Fixable Viability Kit (Biolegend) was used. For compensation, UltraComp eBeads (eBioscience) were used on each day the experiment was performed.

### Staining of samples for flow cytometry

To stain PBMC samples, frozen vials were thawed in a 37°C water bath, washed with FACS Buffer (PBS plus 1% heat-inactivated fetal bovine serum [FBS]), and resuspended in plain PBS at room temperature. The cells were aliquotted into two separate 12 x 75 mm tubes (BD Falcon, Franklin Lakes, NJ): one for staining with the T cell panel of antibodies, and the second for the B cell panel. The cells were washed once with plain PBS, then, stained with Zombie UV dye for 15 minutes in dark at room temperature. The staining was stopped by washing with FACS Buffer, and then, the samples were incubated with mouse IgG at 4°C for at least 5 minutes. Without washing, a cocktail of T or B cell panel of antibodies was added, and the samples were incubated in dark for 30 minutes at 4°C [31,32]. After the incubation, the samples were washed twice with the FACS Buffer, resuspended in the same buffer, and were kept on ice in the dark, and read immediately by a flow cytometer. As a quality control, day 7 post-vaccinated sample from one of the five volunteers who had received the influenza vaccine was stained with T and B cell panels of antibodies in each experiment. Data acquisition was performed with a BD Fortessa flow cytometer equipped with 4 lasers (red, blue, violet, and UV) and 18 detectors using FACSDiVA acquisition software. Data analyses were performed using FCS Express 4 (DeNovo Software).

### Enzyme-linked immunosorbent assay (ELISA) for the measurement of serum anti-HPV16 or -HPV18 antibodies

The production of VLP has been described elsewhere [28,33]. Nunc Maxisorp (Thermo Scientific) flat-bottom 96-well plates were coated with 2.7 μg/mL of L1-based VLP of HPV16 or HPV18 in PBS. The coated plates were washed in Washing Buffer (PBS, pH 7.0 with 1.7 M NaCl, 30 mM Na_2_HPO_4_, 7 mM KHPO_4_, and 0.25% Tween-20 [Sigma Aldrich]), and the wells were blocked with Blocking Buffer (PBS with 4% dry milk and 0.2% Tween-20) for 1.5 hours. After washing, the sera were two-fold serially diluted in the Blocking Buffer, including standard, and positive and negative control sera. After incubating for 1 hour at room temperature on a shaker, the plates were washed, and horseradish peroxidase (HRP)-conjugated goat anti-human IgG antibody (KPL, Gaithersburg, MD) was dispensed. The plates were incubated for 1 hour at room temperature on a shaker, washed, and the substrate (3, 5, 3’, 5’-tetramethylbenzidine [TMB]) (KPL) was added. After incubating for 25 minutes at room temperature, 0.36 N of H_2_SO_4_ (Sigma Aldrich) was added, and the measurements were performed at 450 and 620 nm with a plate reader (SpectraMax M5, Molecular Devices, Sunnyvale, CA). For those with no detectable levels of antibodies were assigned an arbitrary titer value of 4 ELISA Unit (EU) /ml. The cutoffs for HPV16 and HPV18 ELISA were 8 and 7 EU/ml, respectively.

### Statistical analysis

Statistical analyses were performed using GraphPad Prism 6. Paired, one-tailed Wilcoxon rank sum analyses were performed to compare between the percentages of CXCR5^+^CD4^+^ cell subsets or memory B cells at different time points post-vaccination and those from the pre-vaccinated time points. The analyses were performed between the pre-vaccinated time point and D1, D7, D14, or D30 within the vaccine group. The same analyses were also performed between M6 (pre-third vaccination) and D2 post-third vaccination, D7 post-third vaccination, or M7. To compare the statistical differences between Gardasil and Cervarix for different subsets of CXCR5^+^CD4^+^ cells, for memory B cells, and for antibody titers, two-tailed Wilcoxon rank sum analyses were performed. Spearman rank correlational analyses were performed on fold-increase in CXCR5^+^CD4^+^ Tfh-like cell subsets at D7 vs fold-increase in antibody titers at D30 and M7, and fold-increase in memory B cells at various days post-immunizations. It was also performed on percentages of memory B cells at D30 vs antibody titers at D30.

## Results

### Detection and characterization of circulating CXCR5^+^CD4^+^ Tfh-like subsets in influenza vaccine recipients

**Fig 1:**
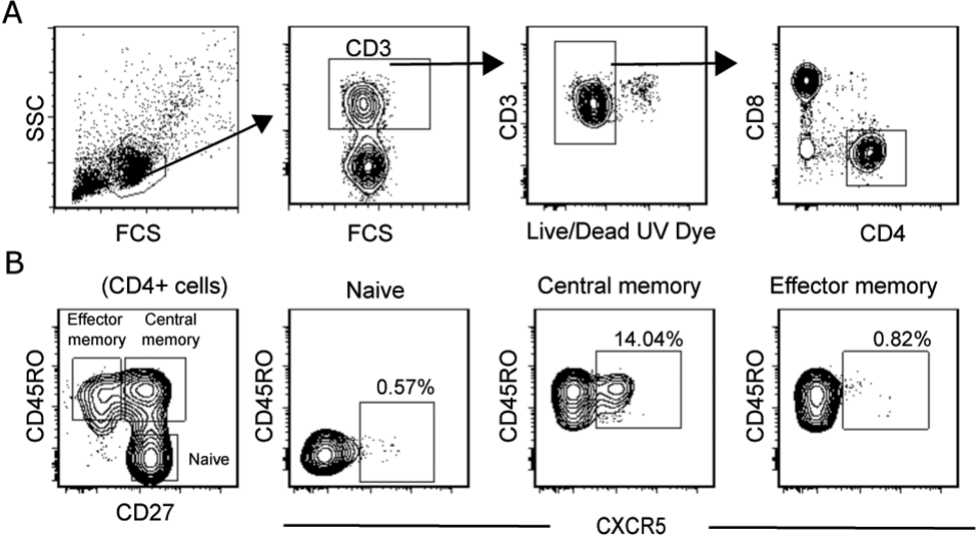
Gating strategy to identify CXCR5^+^ CM cells. (A) Gating scheme used to select live CD3^+^ CD4^+^ cells. (B) Live CD3^+^ CD4^+^ cells were used to identify naive, central memory, and effector memory cells, and CXCR5 expression on these cellular populations was examined.

According to the classification proposed by Ueno and colleagues, nine subsets of circulating CXCR5^+^CD4^+^ Tfh-like cells could be identified [19] (Table 1). Although these circulating Th cells functionally resemble Tfh cells found in lymphoid organs, as they can provide help to B cells, the phenotype does not [11,19]. However, based on the classification proposed by Ueno and colleagues, and for clarity, we will be referring to different subsets of circulating CXCR5^+^CD4^+^ cells as Tfh1-like (CXCR3^+^CCR6^-^), Tfh2-like (CXCR3^-^CCR6^-^), or Tfh17-like (CXCR3^-^CCR6^+^) cells for the remainder of the study. We used the classification shown in Table 1 as a guideline to identify different subsets of circulating CXCR5^+^CD4^+^ cells. The gating strategy we used to identify CXCR5^+^ cells is shown in Fig 1. As expected, we found that the great majority of CXCR5^+^ cells were found within CD45RO^+^CD27^+^ central memory cells (CM). We first examined PBMC samples isolated from the five volunteers who had received the 2013–2014 influenza vaccine to identify the subsets of circulating CXCR5^+^CD4^+^ Tfh-like cellular populations as control experiments to determine whether different subsets could be identified (S2A Fig). Tfh1-, 2-, and 17-like subsets could be delineated using CXCR3 and CCR6 as markers, and Tfh17-like subset was the lowest in frequency (S2A Fig). We also found ICOS was greatly induced in the Tfh1-like subset at day 6 post-influenza vaccination, and that these cells also expressed PD1 (S2A and S2B Fig). Other subsets were not induced in response to the vaccination (S2B Fig). We examined the expression level of CCR7 using the post-vaccination samples (S2C Fig), which contained the PD1/ICOS double positive cell population. The median fluorescent level of CCR7 in naive CD4, CXCR5^+^ CM, PD1/ICOS double negative, PD1^+^ICOS^-^, and PD1/ICOS double positive cell populations were examined. As reported previously, CXCR5^+^ CM cells had a lower CCR7 expression level than that of naive CD4 cells (S2C Fig) [16]. Similarly, we found that the PD1/ICOS double negative cell population had the highest CCR7 expression when compared to the PD1^+^ICOS^-^, and PD1/ICOS double positive cell populations [15]. However, we found that PD1/ICOS double positive cells had higher CCR7 expression than that of PD1^+^ICOS^-^ cells (S2C Fig). Overall, these findings showed that we could identify different subsets of circulating Tfh-like cells.

**Table 1:** Classification of circulating Tfh-like cell subsets.

(CD45RO^+^CXCR5^+^ CD4^+^CD3^+^ cells)	PD1, ICOS, CCR7 expression		
Tfh1-like (CXCR3^+^ CCR6^-^)	PD1^++^ ICOS^+^ CCR7^low^	PD1^+^ ICOS^-^ CCR7^int^	PD1^-^ ICOS^-^ CCR7^Hi^
Tfh2-like (CXCR3^-^ CCR6^-^)	PD1^++^ ICOS^+^ CCR7^low^	PD1^+^ ICOS^-^ CCR7^int^	PD1^-^ ICOS^-^ CCR7 ^Hi^
Tfh17-like (CXCR3^-^ CCR6^+^)	PD1^++^ ICOS^+^ CCR7^low^	PD1^+^ ICOS^-^ CCR7^int^	PD1^-^ ICOS^-^ CCR7 ^Hi^

This classification is based on the model proposed by Schmitt *et al* [19].

### HPV vaccines induced circulating PD1^+^ICOS^+^ Tfh1-like cells

Using the gating scheme shown in Fig 1, we examined samples from the HPV vaccine recipients collected at pre-vaccinated, days 1, 7, 14, and 30 post-first vaccination time points, as well as from M6 (pre-third vaccination), and days 2 and 7 post-third vaccination, and M7. Of note, pre-vaccination time points had the mean of -35 and -58 days for Gardasil and Cervarix, respectively (S1 Table). The percentages of CM cells in either of the vaccine group did not change over time when compared to the pre-vaccination time point (S3A Fig). When we examined the percentages of CXCR5^+^ cells within the CM cell population, percentages increased at days 7, 30, and M6 for the Gardasil recipients but not for the Cervarix recipients (S3B Fig). Neither the percentages of CM cells nor the CXCR5^+^ CM cells were significantly different between the two vaccine groups at the corresponding time points.

**Fig 2:**
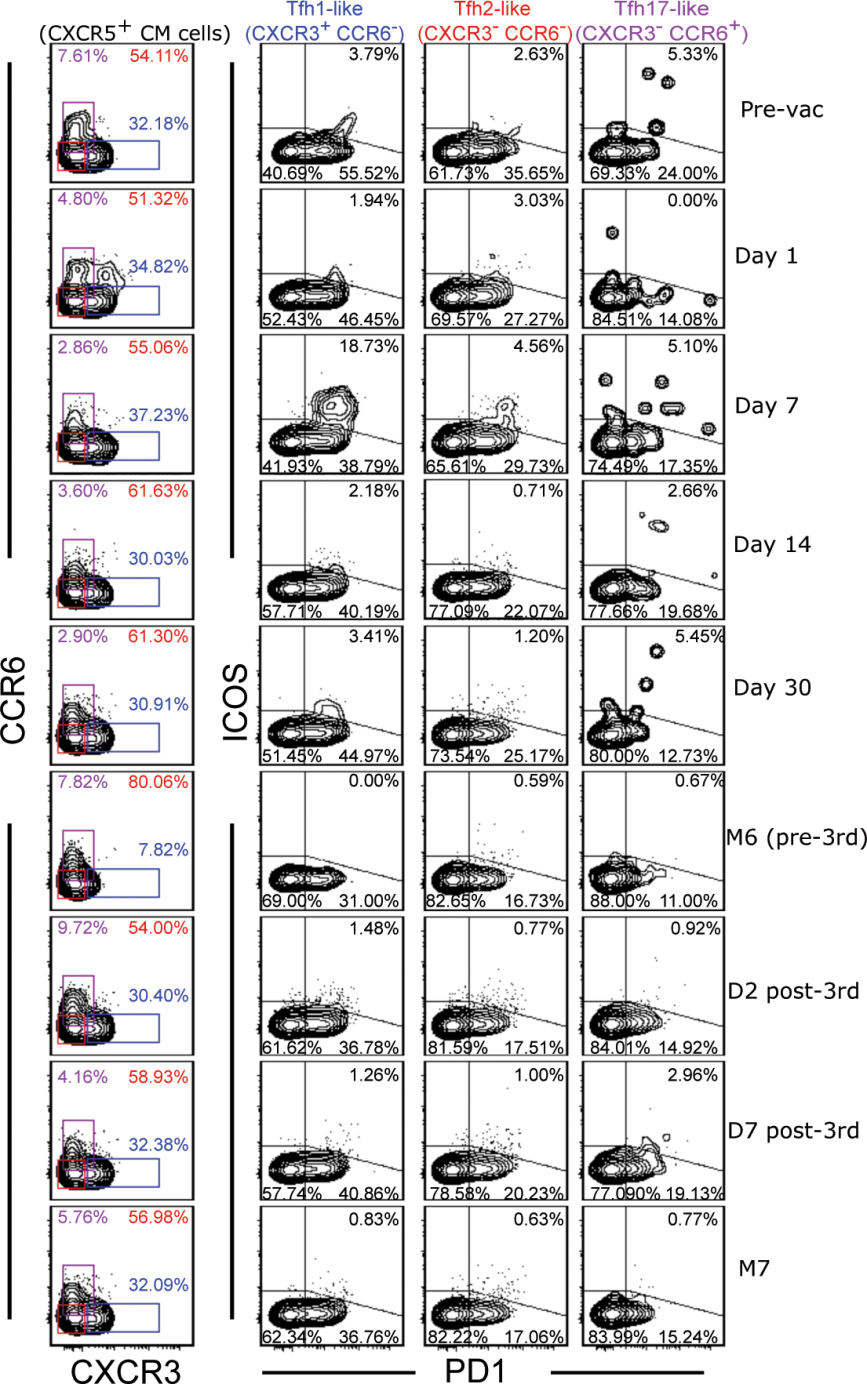
Longitudinal analysis of circulating Tfh1-, Tfh2-, Tfh17-like subsets and their PD1 and ICOS expression. One of the HPV vaccine samples (Gardasil) is shown. The contour plots on the left most column show the expression of CXCR3 and CCR6 on CXCR5^+^ CM cells in samples collected from the indicated time points that are shown on the right. These two markers were used to identify Tfh1-like (blue), Tfh2-like (red), and Tfh17-like (purple) subsets. The percentages of each of these subsets are shown inside the plots. The second, third, and fourth columns of plots show the expression of PD1 and ICOS in each of the Tfh-like subset. In the upper right quadrants, the percentages of PD1^+^ ICOS^+^ Tfh-like cells are shown. The percentages of PD1^+^ ICOS^-^ and double negative cells are indicated in the lower right and lower left quadrants, respectively.

We then delineated the CXCR5^+^ CM cell population into the three subsets using CXCR3 and CCR6 as markers (Fig 2, left most column), and examined the expression of PD1 and ICOS in these circulating Tfh-like subsets (Fig 2). Among them, Tfh17-like cell population was found to be the lowest in frequency. Also, we did not consistently observe CXCR3/CCR6 double positive cells. However, this population is not included in the classification (Table 1). The quadrant gates were used to identify PD1/ICOS double positive cells (upper right quadrant), PD1^+^ICOS^-^ cells (lower right quadrant), and the double negative cells (lower left quadrant) (Fig 2). ICOS single positive cells (upper left quadrant) were not consistently observed in any of the Tfh-like subsets; therefore, we did not pursue this population. Furthermore, when we collectively examined the CCR7 expression on samples from D7 post-first vaccination from both vaccine groups (S4 Fig), we found similar results as those for the influenza samples in S2C Fig.

**Fig 3:**
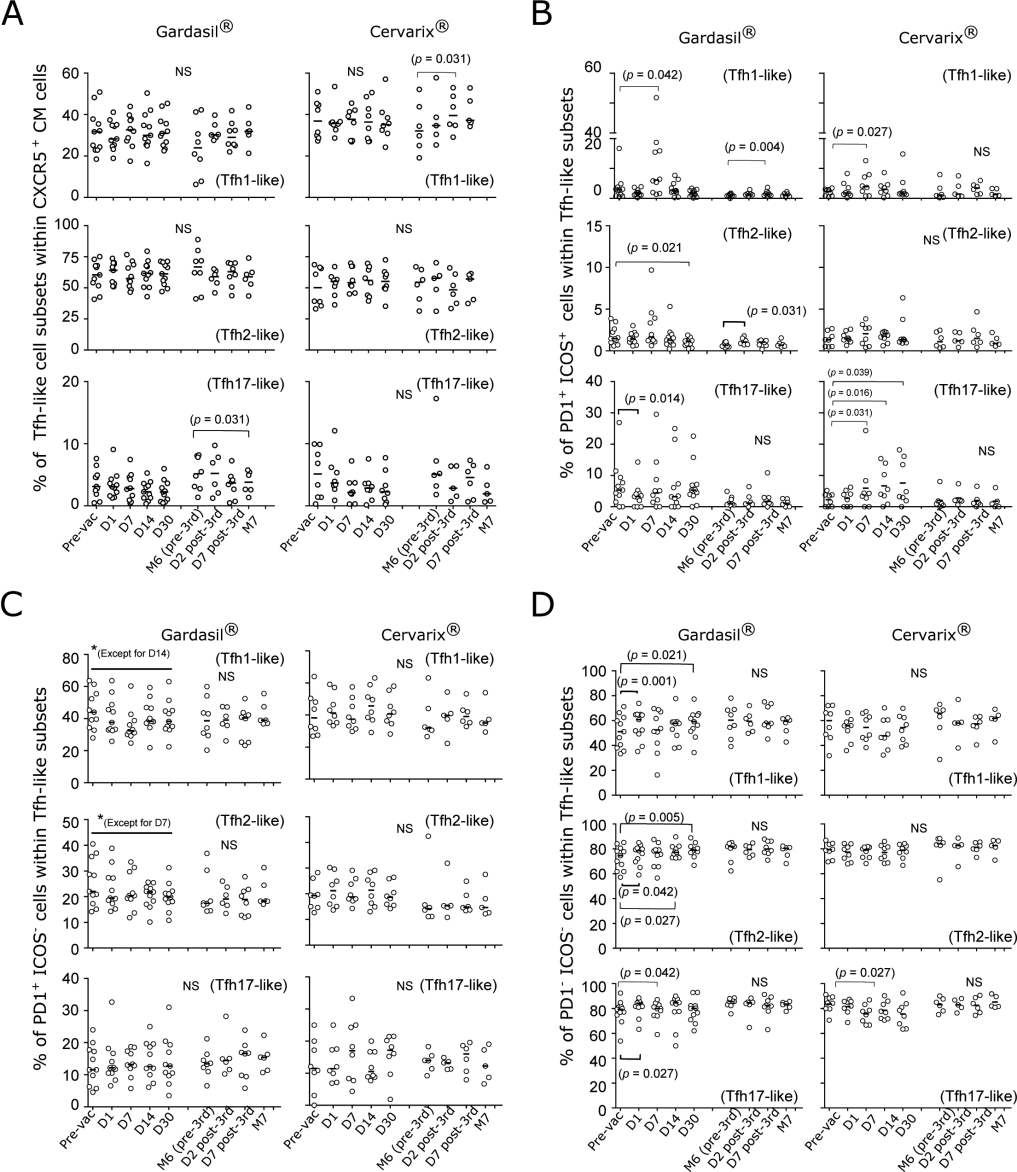
Frequency distributions of different subsets of circulating Tfh-like cells. (A) Percentages of circulating Tfh1-, Tfh2-, and Tfh17-like subsets from the Gardasil and Cervarix groups are shown. (B) Frequencies of PD1/ICOS double positive cells, (C) frequencies of PD1^+^ ICOS^-^ cells, and (D) frequencies of double negative cells within each subset were plotted over time. N = 10–11 (pre-vac to D30) and N = 6–8 (M6 to M7) for Gardasil. N = 8 (pre-vac to D30) and N = 5–6 (M6 to M7) for Cervarix. Paired, one-tailed Wilcoxon rank sum analyses were performed between the pre-vac time point and each of the post-vaccination time point for the first vaccination. For the third vaccination (M6 to M7), M6 was used as the baseline for the analyses. Black horizontal bars indicate the medians. For the comparison of the two vaccine groups described in the “HPV vaccines induced circulating PD1^+^ICOS^+^ Tfh1-like cells” section, paired, two-tailed Wilcoxon rank sum analyses were performed.

We plotted the frequency distributions of different circulating Tfh-like subsets from the two vaccine groups (Fig 3A), the distributions of PD1/ICOS double positive cells (Fig 3B), PD1^+^ ICOS^-^ cells (Fig 3C), and the double negative cells (Fig 3D) as a function of time. It has been reported that the frequency of CXCR3^+^ Tfh1-like cells increased, while it decreased for CCR6^+^ Tfh17-like cells at D7 post-influenza vaccination [18]. In the HPV vaccine cohorts, there were no significant differences in the percentages of Tfh-like subsets compared to the pre-vaccination time points after the first vaccination (Fig 3A). However, after the third dose of the vaccines between M6 (pre-third vaccination) and M7, the frequency of Tfh17-like subset decreased for the Gardasil group, while the frequency of Tfh1-like subset increased for the Cervarix group (Fig 3A).

We found that both HPV vaccines induced ICOS in Tfh1-like subset, which peaked at D7 post-first vaccination, and that these cells also expressed PD1 (Fig 2 and Fig 3B, top panels). Unexpectedly, however, we did not observe much of an induction at D7 post-third vaccination, even though the Gardasil group showed a significant difference when compared to the M6 time point. In the Gardasil group, when we compared the frequencies of the double positive cells at D7 post-first and D7 post-third vaccination, the frequency was higher after the first vaccination (*p* = 0.047). The frequencies of the double positive cells in the Tfh2-like subset were not different; however, there was a trend toward increase after the first vaccination in both groups (Fig 3B, middle panels). In addition, this subset was higher at D2 post-third vaccination when compared to the M6 time point for the Gardasil group. The frequencies of PD1/ICOS double positive Tfh17-like subset increased for Cervarix after the first vaccination but only minimal inductions were observed after the third vaccination (Fig 3B, bottom panels).

### The frequencies of circulating PD1^+^ICOS^-^ and double negative cells

When we examined the frequencies of circulating PD1^+^ICOS^-^ cells (Fig 3C), the frequencies of these population of cells decreased after the first vaccination in the Gardasil group, except for the Tfh17-like subset, while there were no changes in the Cervarix group (Fig 3C). For the double negative cell populations (Fig 3D), we also observed some changes in the percentages of these cells in the Gardasil group. However, it should be noted that the fold-changes in either direction were minimal in the majority of the samples for both populations of cells in Fig 3C and 3D.

Collectively, we found that the HPV vaccines induced ICOS expression primarily in the circulating Tfh1-like subset, and it was most noticeable at D7 post-first vaccination. We also observed a trend toward increase in this double positive population in the Tfh2- and Tfh17-like subsets. In addition, although we observed some phenotypic similarities and differences in the subsets between the two vaccine groups, when we compared the frequencies of these cells in Fig 3 at the corresponding time points, we did not observed significant differences.

### A higher frequency of IgD^-^CD38^Hi^CD27^+^ memory B cells was present in the Cervarix recipients at D30 post-first vaccination

**Fig 4:**
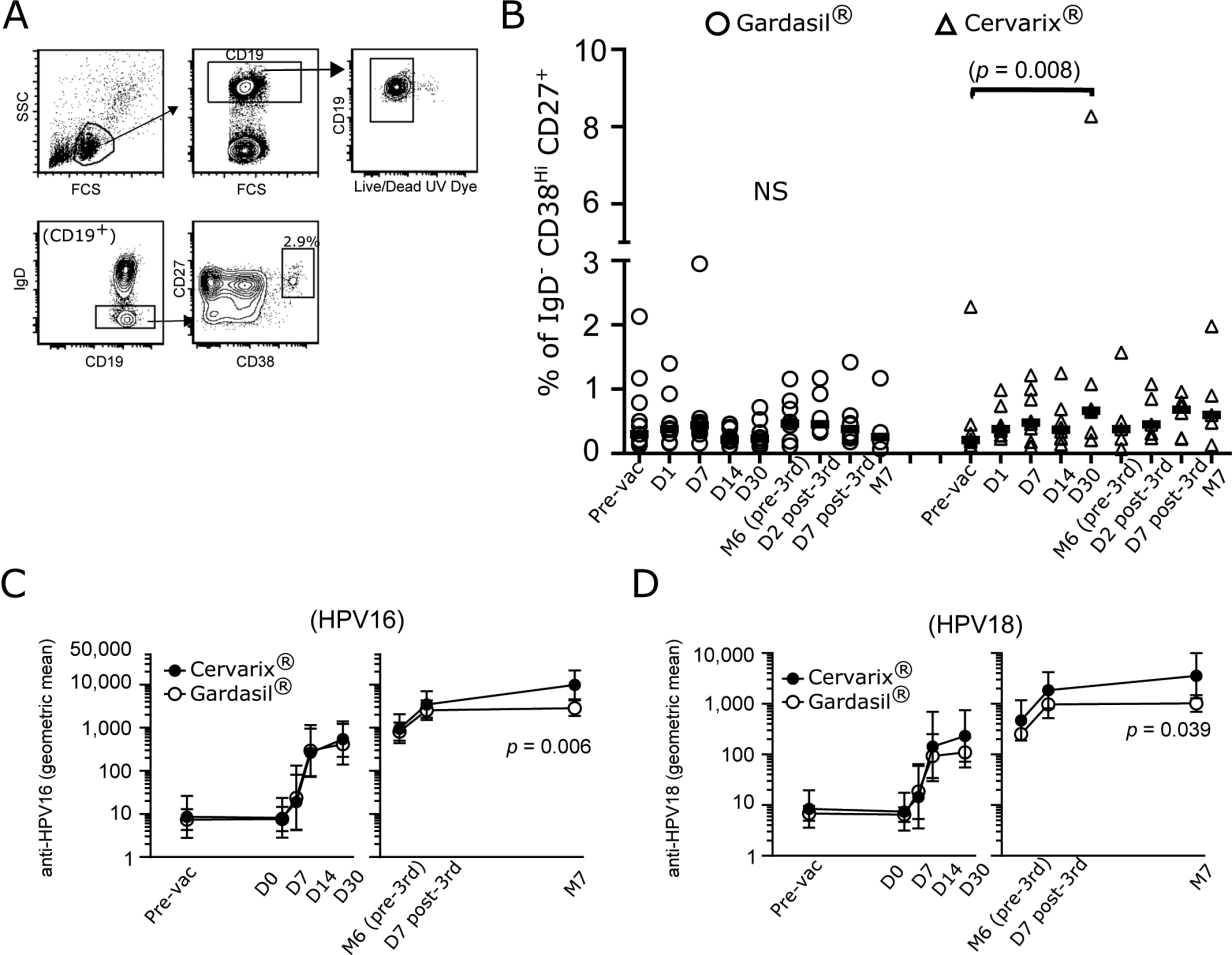
Generation of IgD^-^CD38^Hi^CD27^+^ memory B cells after vaccination. (A) Gating strategy used to identify IgD^-^CD38^Hi^CD27^+^ memory B cells is shown. (B) Percentages of memory B cells in Gardasil or Cervarix immunized recipients were plotted over time. Statistical analyses were performed as described in Fig 3. Black bars indicate the medians. For the comparison of the two vaccine groups described in the “A higher frequency of IgD^-^CD38^Hi^CD27^+^ memory B cells was present in Cervarix recipients at D30 post-first vaccination” section, paired, two-tailed Wilcoxon rank sum analyses were performed. N = 10–11 (pre-vac to D30) and N = 6–8 (M6 to M7) for Gardasil. N = 8 (pre-vac to D30) and N = 5–6 (M6 to M7) for Cervarix. (C and D) ELISA were performed to determine the titers of anti-HPV16 (C) and-HPV18 (D) IgG. Geometric mean antibody titers (EU/ mL) ± 95% confidence intervals in log_10_ scale were plotted as a function of time. The plots on the left show the titers post-first vaccination, and the plots on the right show the titers after the third vaccination. Paired, two-tailed Wilcoxon rank sum analyses were performed. N = 12–15 Gardasil, and N = 8–12 for Cervarix.

As the function of Tfh cells is to provide help to B cells, which leads to class-switching, secretion of antigen-specific antibodies, and memory B cell generation, we measured the levels HPV16- and HPV18-specific IgG, and the frequency of memory B cells. To detect memory B cells, we gated on CD19^+^ IgD^-^ cells to detect CD38^Hi^ CD27^+^ cells (Fig 4A), and plotted the frequencies of these cells over time for both vaccine groups (Fig 4B). We observed significantly higher frequencies of these memory B cells at D30 post-first vaccination when compared to pre-vaccination time point in the Cervarix group only (Fig 4B). Furthermore, when we compared the two vaccine groups at the corresponding time points, higher memory cell frequencies were detected for the Cervarix group at D14 and D30 post-first vaccination (*p* = 0.0234 for D14; *p* = 0.016 for D30). We did not observe statistically significant induction of the memory B cells after the third vaccination in either of the vaccine group.

### Measurement of anti-HPV16 and -HPV18 antibody levels

Although we had previously [28] measured the antibody titers for anti-HPV16 and-HPV18 antibodies for some of the time points we examined in this study, D7 post-first and-third vaccinations, and D14 had not been previously included. Therefore, we measured the titers of anti-HPV16 and-HPV18 IgG in sera from the pre-vaccinated, days 0, 7, 14, and 30 time points by ELISA. We also measured the antibody titers in sera from M6, D7 post-third, and M7. For the serum samples, the D0 (pre-first vaccination) samples were available. In addition, we were able to test all 27 subjects from the study; however, similar to the PBMC samples, samples from some of the time points were not available for some subjects.

The levels of antibodies did not change from the pre-vaccination time point to the D0 time point, suggesting that no major infections had occurred between these two time points. For both vaccines, there were little to no IgG titers at D7 against HPV16 or 18 (Fig 4C and 4D), except for those who had pre-existing titers at the pre-vaccination time point. There were three and two individuals who had pre-existing anti-HPV16 and 18 antibodies in the Gardasil and the Cervarix groups, respectively; indicative of previous exposures to the viruses. By D14 post-first vaccination, anti-HPV16 and-HPV18 antibodies could be detected in all the subjects, nearly reaching the peak levels observed at D30 (Fig 4C and 4D). After the third vaccination, there were increases in the antibody titers at D7 for both vaccine groups, and it further increased at M7 for the Cervarix group, but not for the Gardasil group (Fig 4C and 4D). In this study, geometric mean titers of these antibodies showed that both vaccines induced similar levels of the antigen-specific antibodies to HPV16 and HPV18, except at M7. Of note, among the individuals who had pre-existing HPV antibodies, two from each vaccine group were included in the flow cytometry experiments, and these individuals were included in the analyses for Figs 3 and 4.

### Level of memory B cells at D30 correlated with circulating PD1^+^ICOS^+^ Tfh1-like cells at D7 and with antibody titers at D30

Previous studies reported positive correlations between subsets of circulating CXCR5^+^CD4^+^ cells and antibodies [15,18] as well as with plasmablasts [18]. In contrast, Boswell *et al* did not observed correlations between the percentages of IgG^+^ B cells and circulating CXCR3^-^PD1^+^ICOS^-^CXCR5^+^CD4^+^ T cells in HIV patients [17]. To examine whether there are correlations between different subsets of circulating CXCR5^+^CD4^+^ T cells, and antibodies and memory B cells, we performed Spearman rank correlational analyses. We concentrated our analyses to those subsets that were induced in response to the vaccines, which were primarily those that expressed ICOS. We performed the analyses between fold-change in IgD^-^CD38^Hi^CD27^+^ B cells from different days post vaccination and fold-change in PD1/ICOS double positive Tfh1-, 2-, or 17-like cells at D7 post-first vaccination (Table 2). We found positive correlations at D7 for the Gardasil group, and at D30 for both groups for the Tfh1-like subset (Table 2). We also found a correlation for the Tfh2 subset at D30 in the Gardasil group (Table 2). When we compared these subsets and the changes in the antibody titers, there was a correlation between Tfh1-like cells and anti-HPV18 antibodies at D30 for the Gardasil group but no correlations were found for M7 (Table 3). Also, we have examined for correlations within the third vaccination time period, but we did not find correlations.

**Table 2:** Correlational analyses between PD1^+^ICOS^+^ Tfh-like subsets and memory B cells.

	IgD^-^CD38^Hi^CD27^+^ B cells					
**Gardasil**	**D7 (*r*/*p*)** ^***a***^	**D14 (*r*/*p)***	**D30 (*r*/*p)***	**D2 post-third (*r*/*p)***	**D7-post third (*r*/*p)***	**M7 (*r*/*p)***
PD1^+^ ICOS^+^ **Tfh1 at D7**	(0.693 / 0.031)	NS^*b*^	(0.671 / 0.039)	NS	NS	NS
PD1^+^ ICOS^+^ **Tfh2 at D7**	NS	NS	(0.854 / 0.003)	NS	NS	NS
PD1^+^ ICOS^+^ **Tfh17** ^***c***^ **at D7**	NS	NS	NS	NS	NS	NS
**Cervarix**	**D7 (*r*/*p*)**	**D14 (*r*/*p)***	**D30 (*r*/*p)***	**D2 post-third (*r*/*p)***	**D7-post third (*r*/*p)***	**M7 (*r*/*p)***
PD1^+^ ICOS^+^ **Tfh1 at D7**	NS	NS	(0.862 / 0.008)	NS	NS	NS
PD1^+^ ICOS^+^ **Tfh2 at D7**	NS	NS	NS	NS	NS	NS
PD1^+^ ICOS^+^ **Tfh17 at D7**	NS	NS	NS	NS	NS	NS

Fold changes in different Tfh-like subsets (indicated as Tfh1, Tfh2, and Tfh17) at D7 over the pre-vaccinated time points were compared to the fold changes in the memory B cells at the indicated days (over the pre-vaccinated time points).

^*a*^ *r* indicates correlation coefficient from Spearman rank correlation analyses; *p* indicates *p* value from the Spearman correlational rank analyses.

^*b*^ NS, not significant.

^*c*^ The frequencies of Tfh17 subsets in some subjects were 0% at the baseline; therefore, we could not analyze some of the subjects. For the Tfh1 and 2 subsets, 10 subjects for Gardasil were compared, and it was 8 for Cervarix at D7, 14, and 30. For the other 3 time points, the numbers ranged 5–8 in both groups

**Table 3:** Correlational analyses between PD1^+^ICOS^+^ Tfh-like subsets and antibody titers at D30 and M7.

	Antibodies at D30		Antibodies at M7	
**Gardasil**	**Anti-HPV16 (*r/p*)** ^***a***^	**Anti-HPV18 (*r*/*p)***	**Anti-HPV16 (*r*/*p*)**	**Anti-HPV18 (*r*/*p)***
PD1^+^ ICOS^+^ **Tfh1 at D7**	NS^*b*^	(0.675 / 0.0372)	NS	NS
PD1^+^ ICOS^+^ **Tfh2 at D7**	NS	NS	NS	NS
PD1^+^ ICOS^+^ **Tfh17** ^***c***^ **at D7**	NS	NS	NS	NS
**Cervarix**	**Anti-HPV16 (*r*/*p*)**	**Anti-HPV18 (*r*/*p)***	**Anti-HPV16 (*r*/*p*)**	**Anti-HPV18 (*r*/*p)***
PD1^+^ ICOS^+^ **Tfh1 at D7**	NS	NS	NS	NS
PD1^+^ ICOS^+^ **Tfh2 at D7**	NS	NS	NS	NS
PD1^+^ ICOS^+^ **Tfh17 at D7**	NS	NS	NS	NS

Fold changes in different Tfh-like subsets (indicated as Tfh1, Tfh2, and Tfh17) at D7 over the pre-vaccinated time point were compared to the fold changes in the antibody titers at the indicated time points (over the pre-vaccinated time points).

^a^ *r* indicates correlation coefficient from Spearman correlation rank analyses; *p* indicates *p* value from the Spearman correlational rank analyses.

^*b*^ NS, not significant.

^*c*^ The frequencies of Tfh17 subsets in some subjects were 0% at the base line; therefore, we could not analyze some of the subjects. For the Tfh1- and 2 subsets, 10 subjects for Gardasil were compared, and it was 8 for Cervarix for D30. For M7, the numbers ranged 5–8 in both groups.

**Fig 5:**
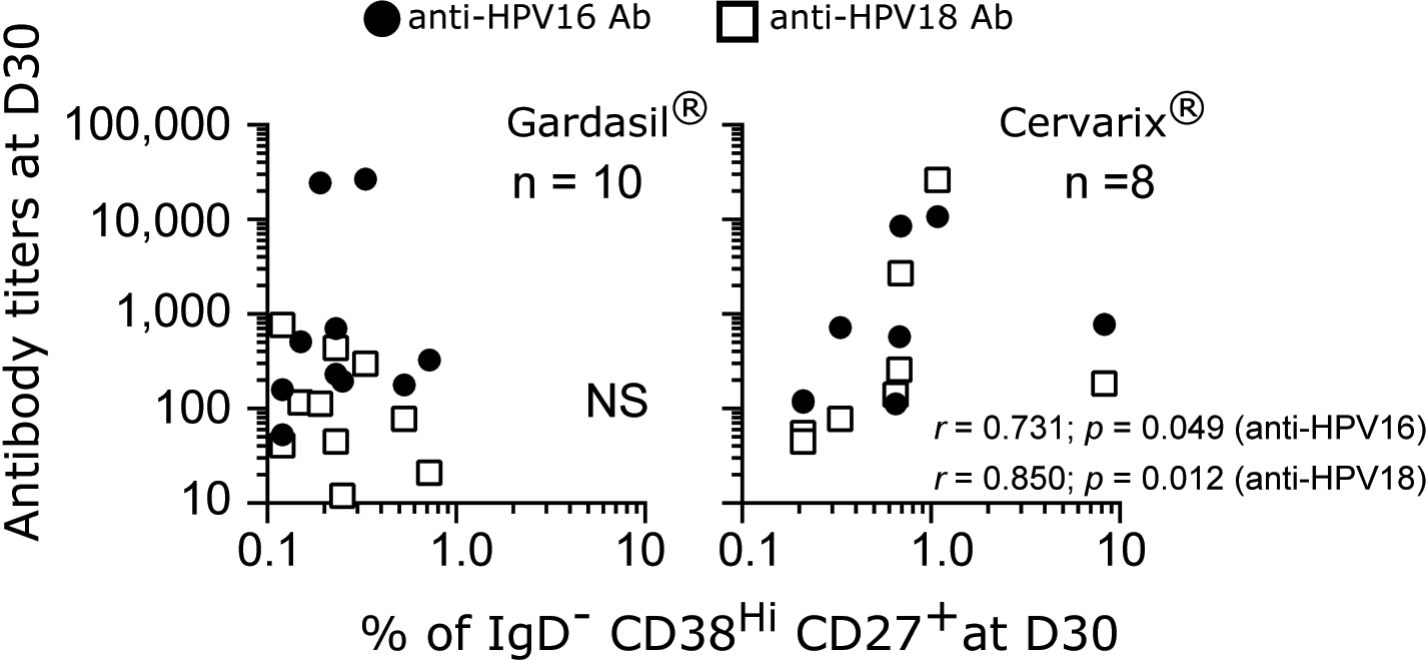
Memory B cell frequencies at D30 correlate with the titers of anti-HPV16 and-HPV18 antibodies. Spearman rank correlational analyses were performed on the percentages of IgD^-^CD38^Hi^CD27^+^ B cells at D30 post-first vaccination with the antibody titers of anti-HPV16 and-HPV18 antibodies from D30. The data were plotted in log_10_ scale for both axes.

We concentrated on D30 post-first vaccination because the majority of these correlations were found at this time point. We performed Spearman rank correlational analyses between the frequencies of memory B cells and the titers of antibodies. We found that the percentages of memory B cells at D30 correlated with both anti-HPV16 and-HPV18 antibodies at D30 for the Cervarix group but not for the Gardasil group (Fig 5). Our analyses showed that memory B cells at D30 for the Cervarix recipients showed good correlations with antibody titers of both HPV16 and 18, as well as with the induction level of PD1/ICOS double positive Tfh-like1 cells.

## Discussion

Based on the seminal works reported by different laboratories, it has been proposed that circulating CXCR5^+^CD4^+^ Tfh-like cells can potentially be used as a biomarker for immune responses to infections and vaccines. As the responses of these Th cells to HPV vaccines or infection have not previously been reported, and that the HPV vaccines provide an excellent model because of their efficacy and immunogenicity, we applied the framework of circulating Tfh-like cells to the HPV vaccine system to explore the potential of monitoring these T cells as a biomarker of immune responses to the vaccines. In addition, because the VRC 902 Study included both of the HPV vaccines, and had an extensive sample collection schedule, it allowed us to examine the immune responses induced by both vaccines using the samples from the same study.

Although circulating CXCR5^+^CD4^+^ T cells can provide help to B cells, their phenotype suggests that they are not fully differentiated Tfh cells, as compared to those found in the secondary lymphoid organs [11,15,17]. However, patients with primary immunodeficiency in ICOS have reduced numbers of circulating CXCR5^+^ CD4^+^ T cells [34]. In addition, formations of GC and memory B cells are also affected. Furthermore, in the absence of signaling lymphocytic activation molecule (SLAM)-associated protein (SAP), a protein that is essential for the formation of stable T/B interaction [35,36], differentiation of Tfh and formation of GC are severely affected in lymphoid organs [37]. Nevertheless, the level of circulating CXCR5^+^CD4^+^ T cells in patients with deficiency in SAP was comparable to that of healthy individuals [16]. Also, *in vitro*, these circulating CXCR5^+^CD4^+^ T cells can provide help to B cells, and produced IL-10, IL-21, and CXCL13; functions associated with Tfh cells [38,39]. Collectively, these studies strongly indicate that circulating CXCR5^+^CD4^+^ T cells represent those Th cells that are either committed to Tfh lineage or related to Tfh cells.

We found CCR7 expression to be higher in the PD1/ICOS double positive cell population than that of PD1^+^ICOS^-^ cells, which was unexpected. This may be because PD1^+^ICOS^-^ cells have the closest transcriptional profile to that of *bona fide* Tfh cells in secondary lymphoid organs [15,17]. The expression of CXCR5 and low CCR7 level on these circulating CXCR5^+^CD4^+^ PD1/ICOS double positive cells or PD1 single positive cells could provide advantage to these cells in positioning themselves in CXCL13 (a ligand for CXCR5)-rich regions of B cell follicles, as low expression of CCR7 will also help to localize these cells in the region, rather than in the T-cell zone [40]. As the interaction with B cells was required for the acquisition of helper activity by these T cells, and that these circulating CXCR5^+^CD4^+^ T cells are not polarized toward particular Th lineages [11], it is plausible that during the interaction with B cells in the follicles that these circulating T cells can acquire the Tfh phenotype *in vivo*.

Similar to influenza vaccine [16,18], we found induction of PD1^+^ICOS^+^ Tfh1 subset after the first dose of the HPV vaccines, which peaked on D7. An interesting study by Suan *et al* showed that Tfh cells were confined to GC during a primary, but not secondary, immune response in mice [41]. However, they did not report whether they examined and detected circulating Tfh-like cells in their study. Furthermore, He *et al* have shown that in mice and humans with SAP deficiency, which leads to severely impaired terminal Tfh cell differentiation in lymphoid organs and GC formation, circulating Tfh-like cells were still detected [16]. Hence, our observations are consistent with their data, in that one would predict to observe the induction of these Th cells in the blood. At this point, although plausible, we do not know whether these induced circulating Tfh-like cells are confined within GC once they have arrived in the secondary lymphoid organs during a primary immune response in humans. In addition, the data reported by Suan *et al* showed that in a recall response, Tfh cells were not confined to GC, and that these Tfh cells were found to scan antigens presented by macrophages that are lining the subcapsular sinuses, and they are the predominant cells responding in a recall response. While this may explain why we did not observe much of an induction after the third dose of the HPV vaccines, it will required further investigations for humans. Alternatively, lack of detection of induction of Tfh-like cells after the third dose of the HPV vaccines could be because the peak level is reached between D2 and D7, and that we may have missed the time point. Although we were not able to examine antigen specificities of the induced circulating subsets of CXCR5^+^CD4^+^ cells in our study, the response kinetics suggested that the inductions occurred in response to the vaccines. Nevertheless, specificity of these T cells needs to be confirmed once tetramers are developed to assure that the induced T cells are HPV specific. Also, *in vitro* functional assay by co-culturing of the circulating Tfh-like subsets with B cells could be performed to examine whether anti-HPV-specific antibodies can be produced. Because of limited availability of the samples, we could not perform the co-culture assay in this study, and to our knowledge, the tetramer system is not available for the detection of HPV L1-specific T cells.

Potentially interesting observations may be that unlike the influenza vaccine, we found both HPV vaccines induced PD1/ICOS double positive Tfh2-like subset. Even though it did not reach statistical significance, there was a trend toward induction. Similarly, the Tfh17-like subset was induced by Cervarix. Induction of these cells was not observed in the influenza vaccinees [18]. It has been suggested that perhaps either both or at least one of the subsets needs to be activated/mobilized for a more efficacious influenza vaccines [13,19]. Because of high success rates associated with the HPV vaccines, the induction of Tfh2-like and/or Tfh17-like subset could be an important factor that influences the efficacy of a vaccine. It is possible that the differences in the Tfh-like subset responses between the influenza vaccine and the HPV vaccines may be because of adjuvants. Studies have been reported that influenza vaccine with adjuvant can influence the diversity and quality of antibody responses [42]. Furthermore, in a murine model, TLR agonists were required to induce responses to influenza vaccine in previously unimmunized mice [43]. However, how the induction of these subsets of Th cells improves and relates to the effectiveness of a vaccine will require further research.

Our data suggested that Cervarix may be more efficient at generating memory B cells after the first vaccination. Although we will need to confirm the data by detecting antigen-specific B cells in the future, this finding is in line with the previous studies that showed Cervarix recipients had more antigen specific B cells than the Gardasil recipients [26,27]. While the frequencies of B cells correlated with the titers of antibodies for Cervarix at D30, it is unclear why they did not correlate for the Gardasil group. Although we observed correlations between different Tfh-like subsets and B cells, and with anti-HPV18. Because of the small sample size of this study, the power of statistical analyses is low, and the biological significance of some of the correlational analyses will need to be confirmed with a larger number of samples. While it has not been determined in the field of HPV what the protective levels of antibody titers are, both vaccines are highly immunogenic, and elicit IgG levels with Cervarix recipients having higher levels of anti-HPV16 and-HPV18 antibodies [26–28]. Our data showed statistically significant differences only at M7. This could be because of the numbers of participants were low in our study. Whether the differences in antibody levels reported in the literature, and the differences in the numbers of B cells we observed for the Cervarix and Gardasil groups could be attributed to the different adjuvants used is uncertain at this point, and requires more work in the future.

Whether three doses of the HPV vaccines are required is an important topic of investigation in the HPV vaccine community. Currently, the field of HPV vaccine appears to be moving toward a two-dose schedule rather than the traditional three-dose schedule in the United States; although there are number of questions that are still required to be addressed [44]. It would be important to examine the types of responses elicited after the second dose of vaccination to understand the immunogenicity of these vaccines.

## Conclusion

In this characterization study, the main findings were that PD1^+^ICOS^+^ Tfh1-like subset was induced on D7 after the primary immunization for both vaccines, Cervarix recipients were associated with higher frequencies of the B cells, and that the antibody titers and B-cell frequencies for Cervarix correlated at D30. Even though the individual responses were variable, we believe that monitoring these circulating Tfh-like subsets early post vaccination could be beneficial in understanding the immunogenicity of vaccines, particularly for those expressing PD1 and ICOS in conjunction with monitoring of memory B cells and perhaps plasma cells. In addition, whenever possible, reagents such as tetramers should be developed and be included in the analysis, as well as reagents to identify antigen-specific B cells. Although we primarily concentrated on the PD1/ICOS double positive cells, there is a need to study whether some of the changes in frequencies we observed in the PD1^+^ ICOS^-^ and the double negative cell populations are biologically meaningful. Because of the small sample size, future studies will need to be extended to different and larger HPV vaccine cohorts with sufficient amount of cells from the appropriate sample collection time points. These studies could contribute to a better understanding of the relationship between circulating Tfh-like cells, memory B cells, and antigen-specific antibodies.

## Supporting Information

S1 Fig Vaccination schedule for Gardasil and Cervarix and the sample collection time points. (A) Schedules of immunization for the two FDA-approved HPV vaccines are shown. (B) The days shown indicate the days at which PBMC samples were prepared from the whole blood. The PBMC samples from these time points were tested in the flow cytometry experiments.
(PDF)

S2 Fig ICOS is induced in CXCR3^+^CCR6^-^ Tfh1-like cell subset after influenza vaccination. (A) Gating strategy used to identify Tfh1-, 2-, and 17-like (indicated as Tfh1, Tfh2, and Tfh17 inside the plots) subsets is shown. Based on these gates, expression of PD1 and ICOS was examined on these cells before (Day 0) and after (Day 6) influenza vaccination. (B) Percentages of PD1^+^ ICOS^+^, PD1^+^ ICOS^-^, and PD1^-^ ICOS^-^ cells in Tfh1-, 2-, or 17-like subset before (Day 0) and after (Day 6–7) the influenza vaccination are shown as line graphs. Paired, one-tailed Wilcoxon rank sum analyses were performed. (C) Median fluorescent intensity of CCR7 was examined in different subsets of Tfh-like cells in post-vaccination samples. Bars indicate medians.
(PDF)

S3 Fig Frequencies of CM cells and CXCR5^+^ cells in HPV vaccine recipients. (A) Percentages of CM cells, and (B) percentages of CXCR5^+^ CM cells were plotted over time. Bars indicate the medians. Paired, two-tailed Wilcoxon rank sum analyses were performed between pre-vac time points with each of the post-vaccination time point. Also, the analyses were performed with M6 and each of the post-third vaccination time points. To compare the two vaccine groups at the respective time points, the same statistical analyses were also performed.
(PDF)

S4 Fig CCR7 expression on different populations of Tfh1-like cells. Median fluorescent intensity of CCR7 was examined on naive CD4^+^ cells, CXCR5^+^ CM cells, double negative cells, PD1^+^ICOS^-^ cells, PD1/ICOS double positive cells, and EM cells in the Tfh1-like subset at D7 post-vaccination from both HPV vaccine groups (N = 18). EM, effector memory. Bars indicate medians. Paired, two-tailed Wilcoxon rank sum analyses were performed. The results from the statistical analyses comparing the CCR7 level among the three groups of Tfh-like cells (PD1/ICOS double negative, PD1^+^ ICOS^-^, and PD1/ICOS double positive cells) are shown.
(PDF)

S1 Table The days on which the samples were collected before and after the vaccinations were determined for each individual participant based on the dates for Day 0. Day 0 is the date on which the participants received the first dose of the vaccines. For the days post-third vaccination, the dates for M6 (pre-third) was used as the starting date.
(DOCX)
