# Supplementary material for: Circulating CXCR5+CD4+ T Follicular-Like Helper Cell and Memory B Cell Responses to Human Papillomavirus Vaccines
Source: PLoS One. 2015 Sep 2;10(9):e0137195. doi: 10.1371/journal.pone.0137195 (PMC4557948; doi:10.1371/journal.pone.0137195)
Supplement: S1 Fig — (A) Schedules of immunization for the two FDA-approved HPV vaccines are shown. (B) The days shown indicate the days at which PBMC samples were prepared from the whole blood. The PBMC samples from these time points were tested in the flow cytometry experiments. (PDF) [file pone.0137195.s001.pdf]

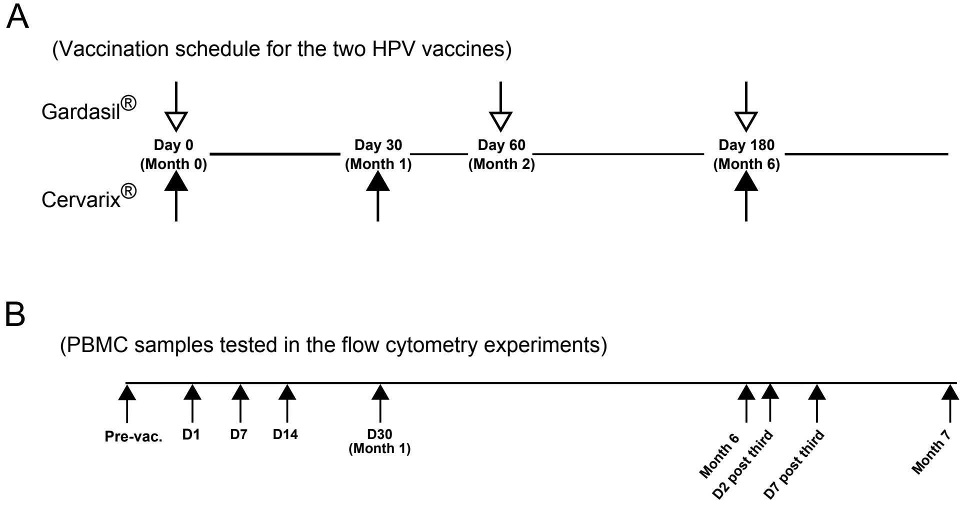
A
(Vaccination schedule for the two HPV vaccines)
Gardasil®
Cervarix®
Day 0
(Month 0)
Day 30
(Month 1)
Day 60
(Month 2)
Day 180
(Month 6)
B
(PBMC samples tested in the flow cytometry experiments)
Pre-vac.
D1
D7
D14
D30
(Month 1)
Month 6
D2 post third
D7 post third
Month 7
