# Supplementary figures and images for: Circulating CXCR5+CD4+ T Follicular-Like Helper Cell and Memory B Cell Responses to Human Papillomavirus Vaccines
Source: PLoS One. 2015 Sep 2;10(9):e0137195. doi: 10.1371/journal.pone.0137195 (PMC4557948; doi:10.1371/journal.pone.0137195)

A

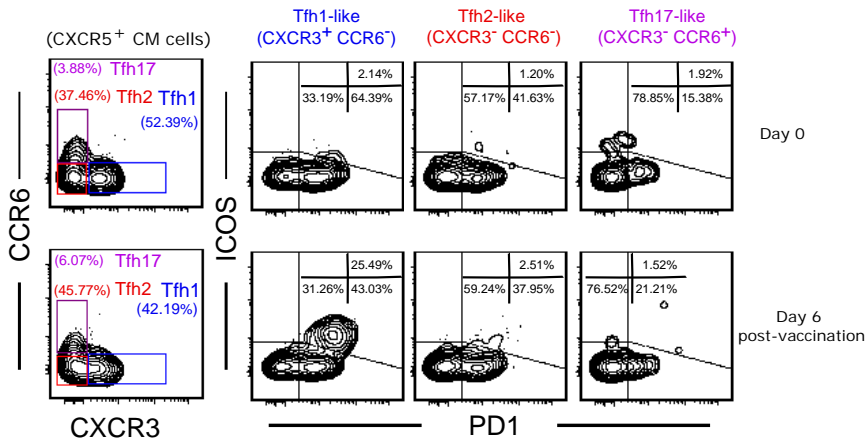

B

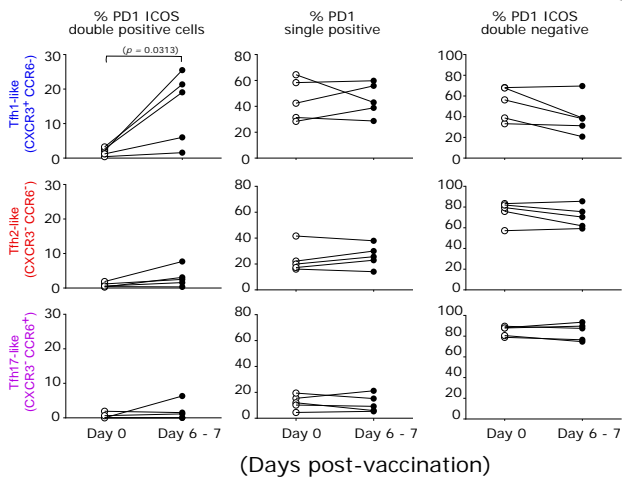

C

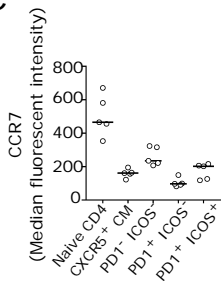

Supplement: S2 Fig — (A) Gating strategy used to identify Tfh1-, 2-, and 17-like (indicated as Tfh1, Tfh2, and Tfh17 inside the plots) subsets is shown. Based on these gates, expression of PD1 and ICOS was examined on these cells before (Day 0) and after (Day 6) influenza vaccination. (B) Percentages of PD1+ ICOS+, PD1+ ICOS-, and PD1- ICOS- cells in Tfh1-, 2-, or 17-like subset before (Day 0) and after (Day 6–7) the influenza vaccination are shown as line graphs. Paired, one-tailed Wilcoxon rank sum analyses were performed. (C) Median fluorescent intensity of CCR7 was examined in different subsets of Tfh-like cells in post-vaccination samples. Bars indicate medians. (PDF) [file pone.0137195.s002.pdf]

A

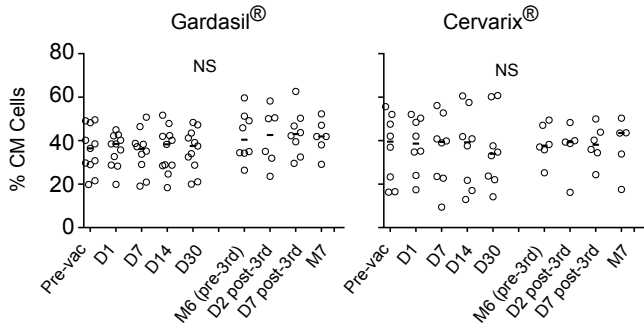

B

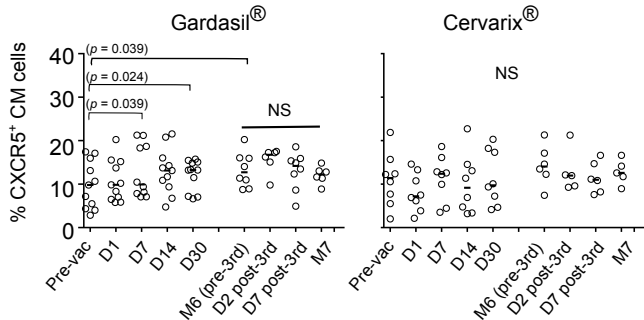

Supplement: S3 Fig — (A) Percentages of CM cells, and (B) percentages of CXCR5+ CM cells were plotted over time. Bars indicate the medians. Paired, two-tailed Wilcoxon rank sum analyses were performed between pre-vac time points with each of the post-vaccination time point. Also, the analyses were performed with M6 and each of the post-third vaccination time points. To compare the two vaccine groups at the respective time points, the same statistical analyses were also performed. (PDF) [file pone.0137195.s003.pdf]

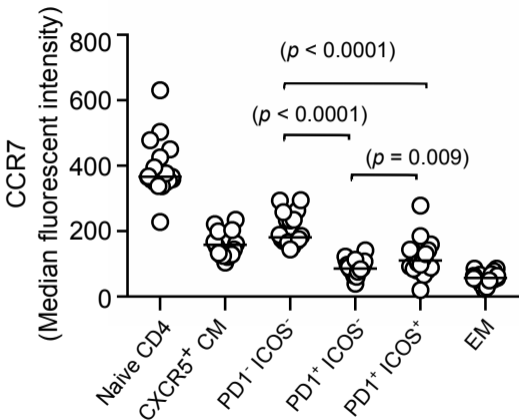

Supplement: S4 Fig — Median fluorescent intensity of CCR7 was examined on naive CD4+ cells, CXCR5+ CM cells, double negative cells, PD1+ICOS- cells, PD1/ICOS double positive cells, and EM cells in the Tfh1-like subset at D7 post-vaccination from both HPV vaccine groups (N = 18). EM, effector memory. Bars indicate medians. Paired, two-tailed Wilcoxon rank sum analyses were performed. The results from the statistical analyses comparing the CCR7 level among the three groups of Tfh-like cells (PD1/ICOS double negative, PD1+ ICOS-, and PD1/ICOS double positive cells) are shown. (PDF) [file pone.0137195.s004.pdf]
