# Supplementary material for: Circulating CXCR5+CD4+ T Follicular-Like Helper Cell and Memory B Cell Responses to Human Papillomavirus Vaccines
Source: PLoS One. 2015 Sep 2;10(9):e0137195. doi: 10.1371/journal.pone.0137195 (PMC4557948; doi:10.1371/journal.pone.0137195)
Supplement: S1 Table — Day 0 is the date on which the participants received the first dose of the vaccines. For the days post-third vaccination, the dates for M6 (pre-third) was used as the starting date. (DOCX) [file pone.0137195.s005.docx]

S1 Table. Days at which the blood samples were collected.

| ***Gardasil^®^*** | **Pre-vaccination** | **Days post-first vaccination** | | | | | **Days post-third vaccination** | | | |
| --- | --- | --- | --- | --- | --- | --- | --- | --- | --- | --- |
|  |  | **D1** | **D7** | **D14** | **D30***^a^* | **M6 (pre-third)***^b^* | **D2** | **D7** | **M7** |  |
| *(Range)* | *(-61 to -21)* | *(1)* | *(6 - 9)* | *(14 - 16)* | *(28 - 36)* | *(157 - 183)* | *(2)* | *(5 - 8)* | *(27 - 57)* |  |
| *Median* | *-34.0* | *1.0* | *7.0* | *14.0* | *28.0* | *168.0* | *2.0* | *7.0* | *41.5* |  |
| *Mean* | *-35.2* | *1.0* | *7.1* | *14.2* | *29.2* | *167.3* | *2.0* | *6.8* | *42.0* |  |
| SD | *10.1* | *0.0* | *1.2* | *0.6* | *2.5* | *7.6* | *0.0* | *1.0* | *12.8* |  |
| ***Cervarix^®^*** |  |  |  |  |  |  |  |  |  |  |
| *(Range)* | *(-152 to -32)* | *(1)* | *(6 - 9)* | *(13 - 15)* | *(28 - 38)* | *(168 - 175)* | *(2)* | *(3 - 8)* | *(31 - 43)* |  |
| *Median* | *-48.5* | *1.0* | *7.0* | *14.0* | *29.5* | *168.5* | *2.0* | *7.0* | *42.0* |  |
| *Mean* | *-58.0****^c^*** | *1.0* | *7.4* | *14.0* | *31.5* | *170.2* | *2.0* | *6.3* | *40.0* |  |
| SD | *38.9* | *0.0* | *1.2* | *0.5* | *4.2* | *3.4* | *0.0* | *1.8* | *5.0* |  |

The days on which the samples were collected before and after the vaccinations were determined for each individual participant based on the dates for Day 0. Day 0 is the date on which the participants received the first dose of the vaccines. For the days post-third vaccination, the dates for M6 (pre-third) was used as the starting date. PBMC samples for Day 0 were not available. *^a^*Samples for D30 were collected prior to the second administration of Cervarix^®^. *^b^*Month 6 samples were collected prior to the third dosage of the vaccines. ***^c^***Two-tailed, paired Wilcoxon rank tests were performed comparing the median dates of collection for Gardasil^®^ and Cervarix^®^. The analyses showed that there were no significant differences at any time points.
